# Thymol in the Treatment of Atrophic Nasal Catarrh

**Published:** 1887-06

**Authors:** 


### THYMOL IN THE TREATMENT OF ATROPHIC NASAL CATARRH.

Ralph W. Seiss, M. D., in the *Medical News* says:

The great difficulty in curing or even permanently improving atrophic nasal catarrh is but too well known to laryngologists, and any agent which promises somewhat better results than those ordinarily obtained deserves the closest study.

Some eighteen months ago I began a series of experiments with thymol, being led to think that it might be of value in nasal diseases from observing its effects when used as a dressing on some general surgical cases. After prolonged and careful trial, the following four standard solutions have been adopted: No. 1. Thymol gr. ss; Alcoholis fʒss; Glycerine fʒss; Aq. dest. q. s. ad. f℥j. M. No. 2. Thymol gr. jss; Alcoholis fʒjss; Glycerine fʒss; Aq. dest. q. s. ad. f℥j. M. No. 3. Thymol gr. v; Alcoholis, Glycerine āā f℥ss; and No. 4 just twice the thymol strength of No. 3. Nos. 1 and 2 have been used in the atomizer, Nos. 3 and 4 are applied by means of a tuft of absorbent cotton on the cotton carrier.

In early cases of atrophic rhinitis where the epithelium is still intact, and the accumulation of discharge is not great—which are characterized by atrophy of the turbinated erectile bodies, with consequent enlargement of the nasal chambers, and by a peculiar shining red hue of the mucous membrane—the No. 1 solution has been used with the most satisfactory results. This form of atrophic catarrh is invariably preceded by the hypertrophic process, and its pathology is briefly described as being a degeneration from pressure and loss of blood supply; the new-formed fibrous tissue of the advanced hypertrophic rhinitis pressing upon the glands and nutrient blood vessels, and so causing atrophy. This disease may exist side by side with the hypertrophic variety: I have never found either large abrasions (shedding of epithelium) or an offensive odor in these cases. Under the thymol treatment used two or three times per week, the nasal secretion becomes more liquid, the mucous membrane regains its normal pink color, and, from the peculiar stimulation of nutrition, the turbinated bodies increase in size, so narrowing the nasal cavities to nearly their normal lumen; and in connection with tonic treatment and careful cleansing of the nasal chambers, the disease has been finally very greatly improved in from three to nine months, in these but slightly advanced cases.

In what I am accustomed to consider *typical* cases of atrophic rhinitis, the following conditions will be found: The patient complains of great, often of almost choking, dryness in the throat, and of accumulation of mucous crusts, particularly in the vault of the pharynx; loss of the sense of smell; and an intolerable sickening odor may or may not be present in greater or less degree. On inspection the mucous membrane is found to be dry and shining, the turbinated bodies nearly or entirely destroyed by atrophy, and erosions (stripping off of epithelium) may occupy nearly the entire mucous membrane. The nasal chambers are greatly enlarged, so that the pharyngeal wall can be plainly seen by anterior rhinoscopy. The surface of the turbinated bodies is rough and irregular from unequal degeneration, and intense congestion from crust irritation is generally present. By posterior rhinoscopy the pharyngeal glandular tissue is found to be almost totally destroyed, and the vault and nasal fossa are blocked by often intensely offensive dark crusts, which may be either dry, or soft and mucous in character, and often completely occlude the Eustachian tubes and posterior nares.

The lower pharynx presents a most peculiar and typical appearance; the posterior wall is dry, often puckered from lack of moisture, and intensely congested, the posterior half arches appear drawn much nearer together than in the normal condition, and on pressure with a probe the mucous membrane is found to be practically resting on the bodies of the vertebræ. Deeply congested and frequently abraded tissues are found on examination with the laryngoscope, and the trachea and bronchi are frequently also diseased.

This advanced stage of turbinated cirrhosis is almost invariably confined to individuals of a so-called "tubercular tendency," or at least to those whose physique is much below par and average resistance. The profound systemic disturbance resulting from typhoid fever, diphtheria, and the like, is frequently the commencement of this disease. But too often rapid intra-nasal atrophy of this variety is the forerunner of phthisis.

In this form of atrophic rhinitis the No. 2 thymol solution is used with the greatest care; the nares are repeatedly sprayed with it until *clean*, and the spray is then driven with a strong atomizer into the mouth and as high up in the pharynx as possible, the treatment being carried out not less than three times a week. After the thymol spray I occasionally touch the deeper abrasions with a solution of nitrate of silver, 100 grains to the ounce of distilled water, and more rarely make applications of properly diluted tincture of *galanga* to the mucous membrane, using a small tuft of cotton on a delieate applicator for the purpose. The pharynx is then painted with the No. 3 or 4 thymol solution, the applicator being gently carried up until the vault is reached. No. 3 is used until it ceases to give pain, when the stronger solution is applied—the applications being made at each sitting to the mucous membrane previously cleansed by the spray.

In all cases of atrophic rhinitis I invariably follow the thymol solution with fluid cosmoline used in an atomizer, and have found this preparation of great value in protecting the mucous membrane and preventing the formation of dried crusts. Patients frequently state that this application gives them greater apparent relief than any other used. The usual tonic and cleansing measures are used in addition, as without them but little permanent improvement will be gained; outdoor exercise and systematic "training" are insisted upon, and strychnia and chloride of ammonium are usually given internally, along with alcohol and careful dieting. Dobell's solution and dilutions of "Listerine" (Lambert) are most frequently ordered as cleansing agents for the patient to use at home, at least twice daily in an atomizer throwing a coarse, strong spray, or by simply snuffing the fluid through the nose; the nasal douche I have entirely abandoned as troublesome and dangerous.

Under the most careful treatment a cure cannot be hoped for, and marked permanent improvement can rarely be obtained in less than seven to nine months; and I have recently seen cases which, although but little improved by six months' treatment, were greatly benefited at the end of a year by the thymol treatment.

There is a third class of cases of so-called *rhinitis cirrhotica*, in which I have found use of thymol particularly beneficial; this form occurs in neglected syphilitic or tubercular children, and occasionally in young adults of the same diathesis. The atrophic process is here very rapid and irregular, the turbinated bodies or their remnants having sometimes an almost honeycomed appearance, or may be entirely destroyed. The amount of secretion thrown off is enormous, the nasal cavities literally *leaking* into the pharanx and down the lips, giving a most disgusting appearance. On inspection the nares are found more or less completely occluded with fluid mucus, abrasions may be numerous and deep, and slight hemorrhages are common. The general appearance of atrophic rhinitis accompanied with congestion obtains: caries of bone and cartilage, and involvement of adjacent sinuses are common. According to Forchheimer, of Cincinnati, ozæna is invariably either syphilitic or tubercular in character, and he states that the bacillus tuberculosis is present in the nasa secretions in all tubercular cases.

This is perhaps the most difficult to cure of any of the forms of atrophic catarrh, but by the use of thymol we have achieved better results than by any other method. The treatment is carried out as for the second class of cases, with the addition of penciling the turbinated bodies with a solution of tincture of *sanguinaria*, *myrrh*, or *galanga* properly diluted with glycerine; the solution selected being applied after careful cleansing with the thymol solutions. Applications of nitrate of silver in the solid form I think positively injurious, from its well-known semi-cauterant and constringing effect; and the insufflation of powdered starch, containing a percentage of silver nitrate, is as unscientific from the standpoint of the chemist, as useless to the rhinologist, the metallic salt being converted by the starch into an inactive oxide, and the starch only serving additionally to occlude the nasal chambers. And drugs applied in the form of powder, with the exceptions of iodoform and boric acid, are the reverse of beneficial in this as in all forms of atrophic rhinitis. Specific treatment, tonics, hygiene and thorough antiseptic cleansing are, of course, insisted upon.

In cases of syphilitic origin, I believe that a practical cure may be obtained by prolonged treatment, although atrophied nasal structures will never be replaced; the tubercular cases rarely improve greatly, and usually die of some intercurrent disease before reaching adult life.

The *causes* of rhinitis cirrhotica are as yet uncertain; why one case of advanced hypertrophic catarrh should remain *in statu quo* throughout the life of the individual, and another rapidly degenerate, has not been demonstrated. Ozæna in infants is probably preceded by an intra-uterine catarrh of the hypertrophic variety, since turbinated cirrhosis *ab initio* is scarcely conceivable by the pathologist. A chronic rhinitis is frequently set up at birth in tubercular or syphilitic children, to degenerate speedily into a true ozæna at the end of a few months of life. I have yet to see a case of advanced turbinated atrophy in even a comparatively otherwise healthy individual, and believe that a "tubercular tendency" is the strongest predisposing factor in the disease, and a weak vitality a necessary soil. Why also we should find in one case the intolerable odor of ozæna, and in another apparently structurally identical no odor whatever, is as yet unproved. I cannot accept the statement of Lefferts, that the odor is due solely to decomposition—from destruction of the serous glands, consequent sticky and tenacious character of the discharges, and damming up and putrefaction of the same within the nasal chambers,—having seen malodorous cases without any considerable amount of pent-up secretion, and observed no odor of consequence in other cases where the nares were completely blocked, the accessory sinuses not being diseased in either case. I think with Mackenzie, of Baltimore, that ozæna from "constitutional causes" is much more likely to give the characteristic stench than that originating from simple inflammatory conditions.

Advanced disease of the *middle ear* is almost invariably found in all cases of turbinated atrophy of long duration; retraction, thickening, and binding down by adhesions of the membrana tympani with consequent deafness, tinnitus aurium, and aural pain, being the most frequent form of disease. Frequently the drum is deformed by cicatrices, and the history of recurrent, acute purulent otorrhœa may be obtained; a marked tendency to these subacute inflammatory attacks of the middle ear, and to the establishment of a chronic purulent otorrhœa, are characteristic of the ear lesions of advanced rhinitis cirrhotica. No improvement can be made in the aural conditions until the nasal disease has been so far as may be "cured," and I have found thymol of especial service in improving the condition of the aural lesions.

Turbinated cirrhosis cannot be considered a curable disease except in the very earliest stages; highly organized structures once destroyed cannot be replaced; and in consequence of the destruction of the serous glands and the great enlargement of the nasal chambers the patient cannot clear his nose by blowing, consequently even a "normal" amount of mucus speedily dries into scales if cleansing measures be discontinued, with the result of fresh crust-irritation and the rapid lighting up of the atrophic process. The best that can be done in advanced cases is to palliate or improve, and cleansing measures must be continued at intervals throughout the life of the individual to insure no active return of the disease.

After having tried almost every form of treatment, a description of which has been published, I now use thymol almost exclusively, and think that by it better results can be obtained in many cases of atrophic catarrh than by any other form of treatment known to me.

